# Peroxisome proliferator-activated receptor γ coactivator-1α (PGC-1α) overexpression alleviates endoplasmic reticulum stress after acute kidney injury

**DOI:** 10.1080/0886022X.2022.2035764

**Published:** 2022-02-27

**Authors:** Hao Pan, Zhizhi Hu, Zhongwen Shao, Yong Ning

**Affiliations:** Department of Nephrology, Tongji Hospital, Tongji Medical College, Huazhong University of Science and Technology, Wuhan, P. R. China

**Keywords:** PGC-1α, acute kidney injury, endoplasmic reticulum stress

## Abstract

**Background:**

Mitochondrial biogenesis dysregulation and enhanced endoplasmic reticulum (ER) stress have been implicated in the progression of acute kidney injury (AKI). However, the interaction between these two events remains poorly understood. This study was designed to investigate the role of peroxisome proliferator-activated receptor γ coactivator-1α (PGC-1α) expression, a key factor in mitochondrial biogenesis, in renal ER stress at 24 h after AKI and the underlying mechanisms.

**Methods:**

Mice were administered recombinant adenovirus encoding murine PGC-1α (100 μl, 1.0 × 10^9^PFU/ml) or vehicle five days before renal ischemia reperfusion (I/R) or sham operation. Twenty-four hours after the operation, kidney and serum samples were collected for evaluation.

**Results:**

We first confirmed that PGC-1α transfection elevated the PGC-1α levels and mitochondrial transcripts in the kidney 24 h after AKI. Then, we found PGC-1α overexpression improved renal function. PGC-1α transfection inhibited AKI-induced ER stress through the unfolded protein response (UPR) pathway, resulting in the suppression of apoptosis *via* both mitochondrial and ER pathways. Further study showed that the expression of mitofusin 2 (Mfn2), an interaction protein between mitochondria and ER, was increased after PGC-1α overexpression. We also found the expression of a novel ER stress regulator, hairy and enhancer of split 1 (Hes1), was decreased after PGC-1α transfection.

**Conclusions:**

Our findings reveal that mitochondrial biogenesis plays an important role in the progression of AKI-induced ER stress and provide useful evidence for research on organelle crosstalk during AKI.

## Introduction

Endoplasmic reticulum (ER) stress is a type of cellular stress that activates a cellular adaptive mechanism, namely, the unfolded protein response (UPR) pathway, to maintain normal ER function and protein homeostasis. The UPR pathway is mainly initiated by ER transmembrane sensors, such as activating transcription factor 6 (ATF6), inositol-requiring enzyme 1 (IRE1) and protein kinase-like endoplasmic reticulum kinase (PERK). However, sustained ER stress may be cytotoxic, leading to apoptosis. It is now clear that ER stress is involved in the pathogenesis of numerous acute conditions that lead to acute kidney injury (AKI) [1]. In these situations, initiating factors such as shortages in oxygen, ATP, nutrients, increased production of reactive oxygen species or chemicals that directly interact with the ER, disrupt ER proteostasis, leading to protein misfolding and accumulation.

Studies have shown that the UPR pathway in the ER also regulates mitochondrial metabolic status [2]. Peroxisome proliferator-activated receptor γ coactivator-1α (PGC-1α) is known as a key regulator of mitochondrial biogenesis and metabolic function in renal tubular cells. It binds with transcription factors to affect different aspects of metabolic processes such as fatty acid oxidation (FAO), oxidative phosphorylation, and reactive oxygen species (ROS) detoxification [3,4]. We and others observed decreased expression of PGC-1α in the kidney and distant organs, which may play an important role in the pathophysiological progression of septic or ischemic AKI [5–7]. However, whether the altered PGC-1α expression affect ER stress *via* UPR pathway or other mechanisms during AKI is still unknown. Therefore, in this study, by using recombinant adenovirus encoding murine PGC-1α (Ad-PGC-1α) to elevate PGC-1α expression, we aimed to explore the effects of PGC-1α on ER stress after AKI as well as the possible underlying mechanisms.

## Materials and methods

### Animals

Male C57BL/6 mice (aged 8–10 weeks, weighing 22–26 g) were purchased from the Animal Experimental Center of Tongji Medical College of Huazhong University of Science and Technology. The mice were housed in an environment with a 12-h light/dark cycle and were given *ad libitum* access to food and water throughout the experimental period. All the animals were cared for in accordance with the Guidance Suggestions for the Care and Use of Laboratory Animals, formulated by the Ministry of Science and Technology of the People's Republic of China.

### Study design

The mice were randomly divided into five groups, namely the sham operation with NS group (sham group), the renal ischemia reperfusion (I/R) with normal saline (NS) group (I/R group), the renal I/R with Ad-PGC-1α group (I/R + Ad-PGC-1α group), the sham operation with Ad-PGC-1α group (sham + Ad-PGC-1α group) and the renal I/R with Ad-control group (I/R + Ad-control group). The sham + Ad-PGC-1α group was established to verify the effectiveness of Ad-PGC-1α. The investigators were blinded to the grouping of the mice.

### Infection with recombinant adenovirus encoding murine PGC-1α

Recombinant Ad-PGC-1α and Ad-control were generated by Biofavor Biotech (Wuhan, China). For *in vivo* infection, 100 μl Ad-PGC-1α (1.0 × 10^9^PFU/ml), Ad-control or NS was administered through the tail vein using a syringe with a 5-gauge needle. The dosage regimen was based on previous literature [8] and our preliminary study.

### Renal I/R procedures

Five days after infection, renal I/R or sham operation was performed according to previous study [9]. Renal I/R was induced by atraumatic vascular clamps placed on both renal pedicles for 45 min. Sham operated mice underwent the same procedure but without vascular clamp placement. The animals were hydrated with warm saline on a heating pad (40 °C), and the body temperature was maintained at 37 °C until the mice fully recovered from anesthesia. Twenty-four hours after renal I/R or sham operation, the mice in each group were euthanized by anesthetic overdose. Kidney and serum samples were immediately collected for later use.

### Renal function measurement

Serum creatinine and blood urine nitrogen (BUN) values are essential parameters for renal function [10]. The concentrations were measured using a commercially available kit (Nanjing Jiancheng Biological Engineering Institute, Jiangsu, China) according to the manufacturer’s instructions.

### Histological examination

Renal tissues were fixed with 4% paraformaldehyde and then embedded in paraffin. Four-micrometer of renal sections were prepared. To evaluate the histological features, renal sections were stained with periodic acid Schiff (PAS) and observed by light microscopy (Olympus, Japan).

### TUNEL staining

TUNEL staining was performed to identify apoptotic cells in renal tissues using commercial reagent (Roche, Germany). Briefly, the tissue sections were deparaffinized and pretreated with 0.1 M sodium citrate, at 65 °C for 30 min and then incubated with TUNEL reagents for 1 h at 37 °C in a humidified, dark chamber. After counterstaining with DAPI, the positive staining was detected by fluorescence microscopy (Olympus, Japan). For quantification, 10 representative fields were selected from each tissue section and the number of TUNEL positive-cells per mm^2^ was evaluated.

### Immunohistochemical staining of hairy and enhancer of split 1 (Hes1)

A modified immunohistochemical staining protocol was performed. Briefly, the kidney sections were deparaffinized and after rehydration, antigen retrieval was performed by incubation with 1 mM EDTA (pH 8.0) at 95–100 °C for 20 min. The tissue sections were then incubated with a blocking buffer, followed by anti-Hes1 (Abcam, Cambridge, UK) at 4 °C overnight. The samples were then incubated with horseradish peroxidase (HRP)-labeled goat anti-rabbit antibody for 50 min, and then, the sections were further incubated with the diaminobenzene (DAB) regent (Dako, Agilent Technologies, Inc.). The slides were observed by light microscopy (Olympus, Japan) equipped with a digital camera. For quantification, 10 representative fields were selected from each tissue section and the number of Hes1 positive dots per mm^2^ was evaluated.

### qPCR analysis

The transcript levels of mitochondrial transcript genes, twinkle mtDNA helicase (Twnk) and mitochondrial transcription factor A (Tfam) were measured by qPCR. Total RNA samples were extracted from kidney using TRIzol reagent (Invitrogen, USA) according to the manufacturer’s instructions. Total RNA was reverse transcribed using reverse transcription (Superscript, Invitrogen, USA). qPCR was performed employing SYBR green PCR Master Mix (Fermentas, Thermo Scientific, USA) with sequence-specific primers. The sequences of the primers are presented in Table 1. All the gene expression values were normalized using β-actin as a housekeeping gene.

**Table 1.** Primers sequences used in the qPCR.

Gene	Forward primer (5'-3')	Reverse primer (5'-3')
Twnk	GACTTTCCACGGACAACAGAG	AGTTCCTTGTCGTCGTCTTCT
Tfam	GGAGGCAAAGGATGATTCGG	TCGTCCAACTTCAGCCATCT
β-actin	CACGATGGAGGGGCCGGACTCATC	TAAAGACCTCTATGCCAACACAGT

### Western blot analysis

Equal amounts of protein samples from mouse kidney were separated by SDS-PAGE, transferred onto polyvinylidene fluoride membranes by electroblotting and incubated at 4 °C overnight with primary antibodies (anti-PGC-1α (Abcam, Cambridge, UK), anti-ATF6 (Santa Cruz Biotechnology, USA), anti-C/EBP homologous protein (CHOP, Santa Cruz Biotechnology, USA), anti- phospho-IRE1α (p- IRE1α, Invitrogen, USA), anti-phospho-PERK (p-PERK, Invitrogen, USA), anti-cleaved Poly (ADP-ribose) polymerase 1 (c-PARP1, Santa Cruz Biotechnology, USA), anti-mitofusin 2 (Mfn2, Abcam, Cambridge, UK), anti-apoptotic protein B-cell lymphoma 2 (Bcl2, Santa Cruz Biotechnology, USA), anti-Bcl2-associated X protein (Bax, Santa Cruz Biotechnology, USA), anti-cleaved caspase 3 (Cell Signaling Technology, USA), anti-caspase 12 (Cell Signaling Technology, USA), anti-Notch1 (Abcam, Cambridge, UK), anti-Hes1(Abcam, Cambridge, UK) and anti-GAPDH (Invitrogen, USA)). Next, the membranes were incubated with horseradish peroxidase–conjugated goat anti–rabbit secondary antibody (Invitrogen, USA) at 25 °C for 1.5 h. The chemiluminescent bands were visualized using a chemiluminescence detection system and quantified using ImageJ software.

### Statistical analysis

All the data were expressed as the mean ± SEM. Normally distributed data were analyzed with one-way ANOVA with Student–Newman–Keuls *post hoc* test. Statistical analyses were performed employing GraphPad Prism 8.0 (GraphPad Software Inc., CA, USA). Significance was set as *p* < 0.05.

## Results

### PGC-1α overexpression promotes mitochondrial biogenesis in the kidney after AKI

**Figure 1.**
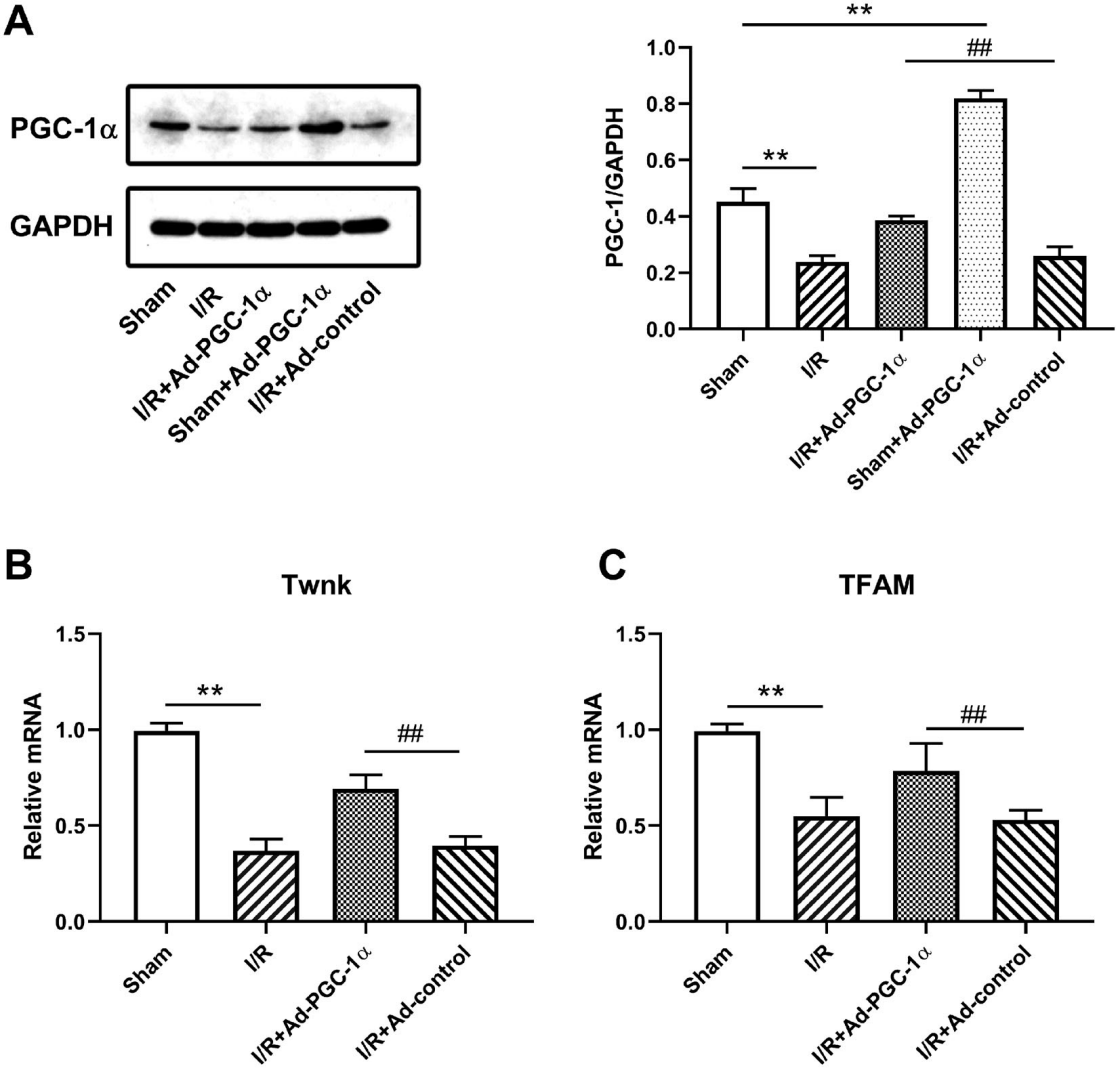
PGC-1α expression and mitochondrial transcripts in kidney. (A) Western blot analysis of PGC-1α expression in kidney. (B, C) qPCR analysis of Twnk and Tfam in different groups. **p < 0.01 *versus* sham group; ^##^*p* < 0.01 *versus* I/R + Ad-control group.

Western blotting was used to investigate the expression of PGC-1α in different groups (Figure 1(A,B)). First, we confirmed that PGC-1α transfection significantly increased the PGC-1α protein levels in the kidneys of sham group (*p* < 0.01). The AKI group exhibited a reduction in PGC-1α expression in the kidney compared with the sham group (*p* < 0.01). Moreover, PGC-1α overexpression increased the PGC-1α protein level in the kidney after AKI compared with I/R + Ad-control (*p* < 0.01). qPCR was used to investigate the key molecules of mitochondrial DNA copy number in the different groups (Figure 1(B,C)). We found that the mRNA levels of Twnk and Tfam were both reduced after AKI (*p* < 0.01), but increased after PGC-1α transfection (*p* < 0.01).

### PGC-1α overexpression protects renal function during AKI

**Figure 2.**
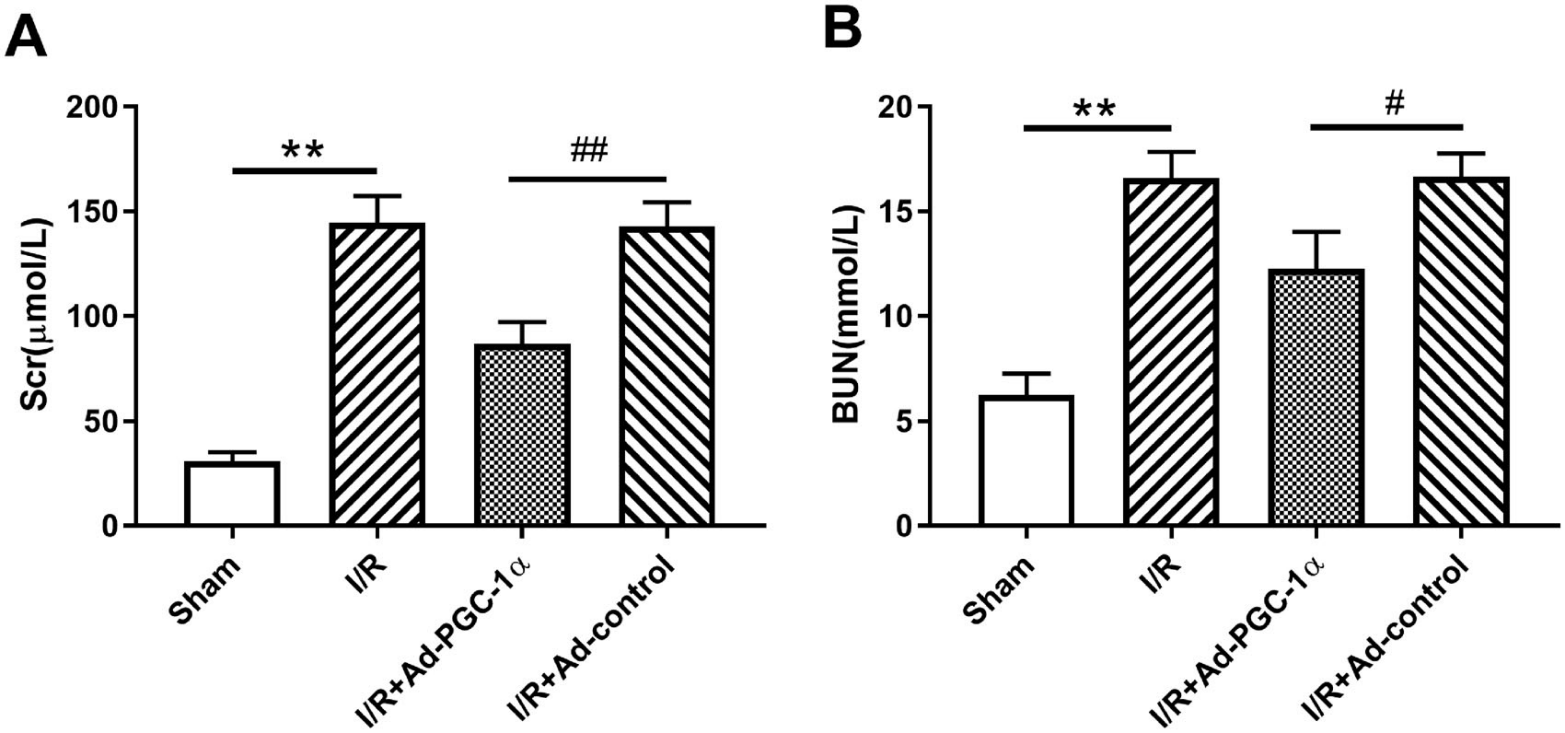
PGC-1α overexpression alleviated kidney function and inflammation. (A) The level of serum creatinine in different groups. (B) The level of serum urine nitrogen in different groups. **p < 0.01 *versus* sham group; ^##^*p* < 0.01 *versus* I/R + Ad-control group.

We evaluated the serum creatinine and urine nitrogen levels in the different groups to assess renal function. As shown in Figure 2(A–B), the I/R group showed an elevated serum creatinine (144.33 ± 13.05 *vs.* 30.67 ± 4.51 μmol/l, *p* < 0.01) and urine nitrogen (16.59 ± 1.25 *vs.* 6.26 ± 1.01 mmol/l, *p* < 0.01) levels, indicating impaired renal function. However, the mice in the Ad-PGC-1α group showed significantly lower serum creatinine (86.67.67 ± 10.59 μmol/l, *p* < 0.01) and urine nitrogen (12.27 ± 1.76 mmol/l, *p* < 0.01) levels, indicating a better renal function.

### PGC-1α overexpression ameliorates kidney histopathological changes

**Figure 3.**
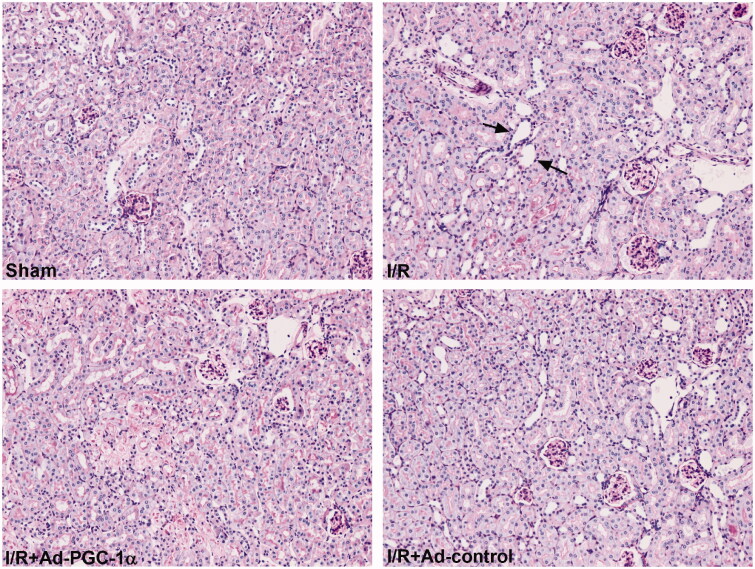
PGC-1α overexpression attenuated renal tubulointerstitial damage. Histological changes of kidney in each group by PAS (×200). Black arrow: brush border loss and tubular dilation.

At 24 h after AKI, as shown in Figure 3, compared with the sham group, there was widespread tubular necrosis, mass brush border loss and tubular dilation in the I/R group, as detected by PAS staining. The mice in the Ad-PGC-1α group showed milder histopathological changes than those in the I/R + Ad-control group.

### PGC-1α overexpression alleviates ER stress in the kidney after AKI

**Figure 4.**
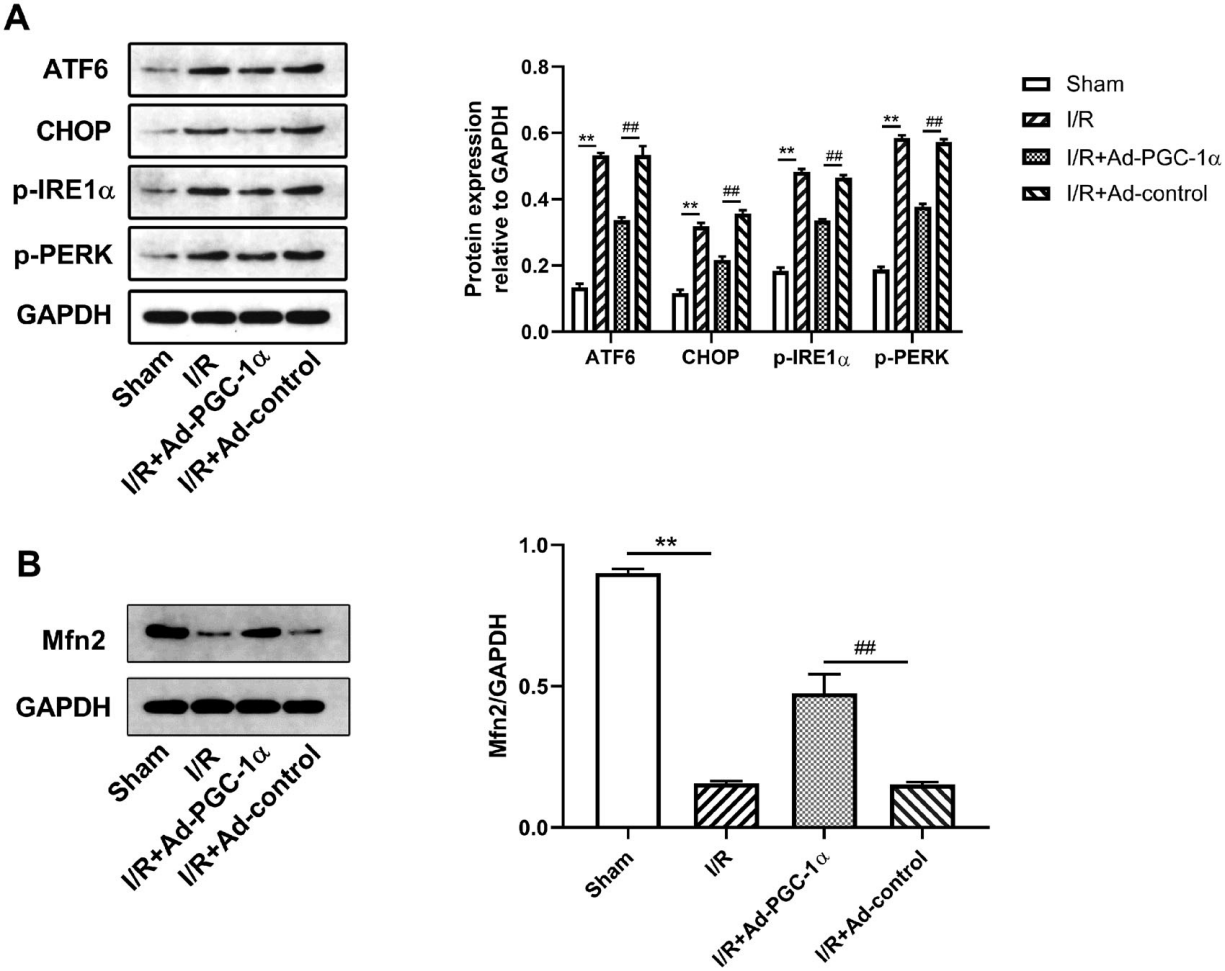
PGC-1α overexpression ameliorates AKI induced ER stress and restores Mfn2 expression in kidney. (A) Western blot analysis of ER stress sensors expression in each group. (C) Western blot analysis of Mfn2 expression in each group. **p < 0.01 *versus* sham group; ^##^ *p* < 0.01 *versus* I/R + Ad-control group.

To evaluate the effect of PGC-1α overexpression on ER stress in the kidney after AKI, we assessed the levels of ER stress markers, including ATF6, CHOP, p-IRE1α and p-PERK. As shown in Figure 4(A), we found that the expression of all four ER stress markers was significantly increased, but decreased after PGC-1α overexpression. Our results indicate that PGC-1α overexpression inhibits ER stress after AKI.

### PGC-1α overexpression increases Mfn2 expression in the kidney after AKI

We tested the level of Mfn2 expression in the kidney by Western blot. As shown in Figure 4(B), we found that after AKI, the expression of Mfn2 was markedly decreased, while PGC-1α overexpression significantly increased Mfn2 expression.

### PGC-1α overexpression inhibits apoptosis in the kidney after AKI

**Figure 5.**
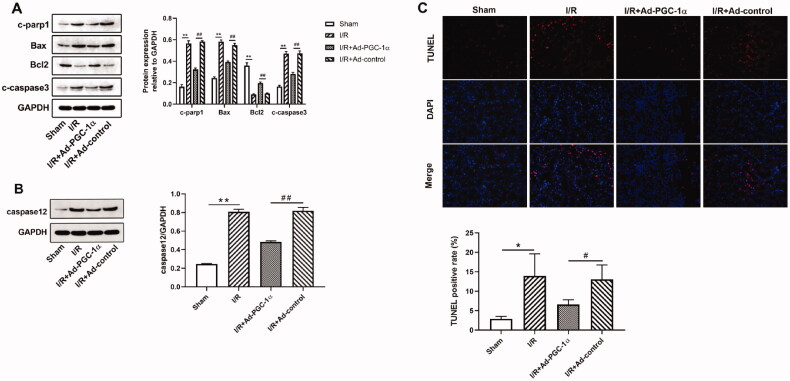
PGC-1α overexpression inhibits apoptosis in kidney. (A) Western blot analysis of apoptosis proteins in different groups; (B) Western blot analysis of caspase 12 in different groups; (C) TUNEL assays in different groups. **p < 0.01 *versus* sham group; ^##^ *p* < 0.01 *versus* I/R + Ad-control group; *p < 0.05 *versus* sham group; ^#^*p* < 0.05 *versus* I/R + Ad-control group.

To examine whether PGC-1α overexpression protects against apoptosis in the kidney after AKI, we assessed the levels of apoptosis related proteins. As shown in Figure 5(A–B), we found that the expression of proapoptotic proteins, such as c-PARP1, BAX and c-caspase 3, and ER associated caspase 12 were significantly increased, while the level of antiapoptotic protein, Bcl2, was decreased in the AKI group compared with the sham group. PGC-1α overexpression significantly decreased the expression of proapoptotic proteins and caspase 12, while increased the expression of antiapoptotic proteins. We further assessed apoptosis by TUNEL staining. As shown in Figure 5(C), TUNEL positive cells were rarely observed in the kidneys of the sham group, but these numbers were increased after AKI. Furthermore, the numbers of TUNEL positive cells in the kidney were decreased after PGC-1α overexpression. Further quantification analysis also confirmed this trend. Together, these findings indicate that PGC-1α overexpression protects against apoptosis in the kidney after AKI.

### PGC-1α overexpression downregulates Notch1 and Hes1 expression in the kidney after AKI

**Figure 6.**
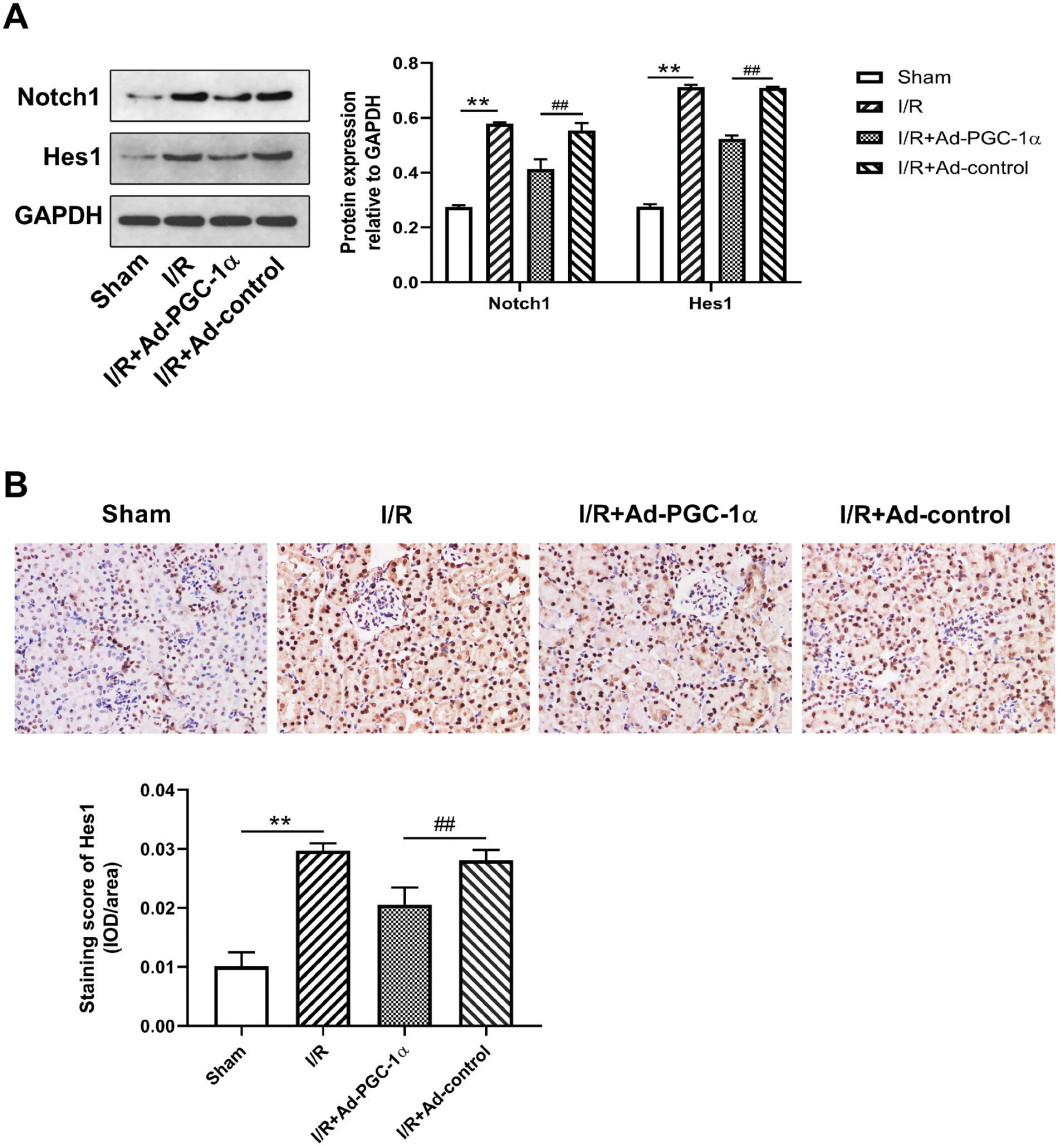
PGC-1α overexpression decreases Notch1 and Hes1 expression in kidney. (A) Western blot analysis of Notch1 and Hes1 expression in different groups; (B) Immunohistochemical staining of Hes1 in different groups. **p < 0.01 *versus* sham group; ^##^ *p* < 0.01 *versus* I/R + Ad-control group.

We evaluated the levels of Notch1 and Hes1 expression in the kidney by Western blot and immunohistochemical staining. As shown in Figure 6(A), we found that after AKI, the expression of both Notch1 and Hes1 was markedly increased, while PGC-1α overexpression significantly decreased the expression of both Notch1 and Hes1. Immunohistochemical staining showed increased Hes1 expression in the cytoplasm of renal tubular cells after AKI (Figure 6(B)). Quantitatively, the numbers of Hes1 positive cells were reduced after PGC-1α overexpression (*p* < 0.01).

## Discussion

Mitochondrial dysfunction and ER stress are two key factors in the pathophysiology of AKI. It is well known that crosstalk between mitochondria and ER may regulate distinct cellular response to certain stimuli in the pathogenesis of many diseases. Mitochondrial biogenesis is an important process for ATP synthesis in the cells. Decreased ATP production caused by mitochondrial dysfunction may affect ER function. In the current study, we found decreased mitochondrial biogenesis and increased ER stress in the kidney at 24 h after AKI. Overexpression of PGC-1α significantly improved mitochondrial function, alleviated ER stress through UPR pathway, inhibited mitochondrial and ER related apoptosis in the kidney, possibly associated with Mfn2 and Hes1 regulation. Our results demonstrate the importance of proper mitochondrial biogenesis to ER stress at 24 h after AKI.

PGC-1α is known as the primary factor in mitochondrial biogenesis, metabolic function and antioxidant gene expression. It activates Tfam to regulate mitochondrial DNA (mtDNA) copy number, which is crucial for mitochondrial biogenesis. Twnk is also involved in the production and maintenance of mtDNA. In the present study, by using Ad-PGC-1α to elevate global PGC-1α expression before AKI, we found an increased Twnk and Tfam expression in the kidney 24 h after AKI, indicating an increased mtDNA copy number and better renal mitochondrial biogenesis. Our further study showed that PGC-1α overexpression resulted in better kidney function and improved kidney histopathological changes at 24 h after AKI, which is consistent with previous studies.

As described, the UPR pathway in the ER plays an important role in the maintenance of ER homeostasis. Moreover, the mitochondrial UPR also functions to optimize ER-mitochondrial interactions [2]. Due to the importance of PGC-1α expression in mitochondrial function, we wondered whether PGC-1α overexpression has any effect on ER stress 24 h after AKI. As a result, we observed that 24 h after AKI, PGC-1α overexpression significantly decreased the expression of major ER stress markers in the UPR pathway, indicating alleviation of ER stress in the kidney.

Numerous studies have indicated that ER stress sensors are associated with the activity of PGC-1α, thereby linking the UPR to metabolic gene programs. In skeletal muscle, PGC-1α is upregulated in response to ER stress and cooperates with ATF6 to mediate an adaptive response to stress [11]. In the liver, persistent ER stress suppresses PGC-1α through ATF6 [12]. In addition, in hepatoma cell lines, ATF6 and PGC-1α activities enhance estrogen-related receptor gamma (ERRγ) expression. In turn, ERRγ interacts with PGC-1α to mediate the transcription of ERR target genes. ATF6 has also been identified as an ERRγ/PGC-1α target, suggesting a feedforward mechanism by which PGC-1α mediates adaptation to stress [13]. In addition to ATF6, previous studies have shown that diabetes-induced ER stress represses PGC-1α through CHOP expression induction [14]. In drug-resistant colorectal cancer cells, PGC-1α is also reported can control mitochondrial biogenesis by regulating ER stress [15]. Together with our results, these findings suggest that there seems to be crosstalk between PGC-1α mediated metabolic processes and ER stress through the UPR pathways in variety conditions, including AKI.

Mfn2 is expressed on both the ER and mitochondria, and serves to tether mitochondria to the ER. The interaction between the ER and mitochondria through Mfn2 plays an important role in the maintenance of mitochondrial fusion and fission, indicating that this interaction regulates the structural homeostasis of both organelles [16]. In our study, we found that Mfn2 expression was decreased after AKI, but significantly increased after PGC-1α overexpression. In fact, previous studies have suggested that Mfn2 and altered mitochondrial dynamics are upstream of ER stress such that a reduction in Mfn2 triggers ER stress [17]. PGC-1α plays a key role in mitochondrial dynamics. PGC-1α also activates the PINK1/Parkin mitophagy pathway, which is involved in ubiquitination of Mfn2 [18–20]. The PERK and ATF6 pathways have also both been suggested to affect Mfn2 expression through PGC-1α [21]. Similar with these reports, our study provides further evidence that mitochondrial biogenesis associated mitochondrial dynamics may affect ER stress *via* altered Mfn2 expression in the kidney after AKI.

As described, PGC-1α plays a key role in regulating ROS production. which have been reported can induce ER stress, leading to apoptosis. Thus, enhanced mitochondrial biogenesis has been observed to markedly rescue cells from ER stress-mediated death by abrogating ROS production [22,23]. In the current study, we found PGC-1α overexpression significantly reduced renal apoptosis at 24 h after AKI. In addition to the mitochondrial pathway, we found that caspase 12 activation was also inhibited, the latter is specific for ER stress-induced apoptosis [24]. Previous reports have been found that reduced ROS generation may suppress caspase 12 expression [25,26]. Although we did not detect ROS production, obviously, PGC-1α overexpression induced better mitochondrial function decreased ROS production in the kidney at 24 h after AKI. Together with these studies, our results suggest that PGC-1α overexpression can inhibit renal apoptosis through both mitochondrial and ER pathways, which also reflected that it alleviated renal ER stress after AKI. More detailed mechanisms need to be further studied.

Interestingly, in the current study, by using Western blot and immunohistochemical staining, we found that Hes1 expression increased in the kidney 24 h after AKI, but decreased after PGC-1α overexpression. Our result is similar to previous studies, which found that Hes1 expression in the kidney reaches a peak at 24 h after AKI and that prolonged Hes1 activation may cause AKI-induced inflammation and apoptosis [27–29]. Hes1 is activated by both canonical and noncanonical pathways, and Notch represents one of the prominent canonical pathways [30]. In our study, we found that the expression of Notch1 and Hes1 exhibited the same trend, suggesting that Hes1 activation is derived from the activated Notch1/Hes1 pathway in response to AKI. Notch activation plays critical roles in renal fibrosis development [31]. Hes1 was reported to bind directly to the Ppargc1a promoter region and inhibit PGC-1α expression in animal models of chronic kidney disease (CKD). In the same study, overexpression of PGC-1α in tubular cells almost completely prevented Notch-induced renal fibrosis *in vivo* [32]. Here, we also observed a similar phenomenon in the AKI model induced by global PGC-1α overexpression. The results indicated that PGC-1α overexpression provides sufficient metabolic input and can reduce Notch1/Hes1 pathway activation, thereby alleviating AKI induced kidney inflammation, apoptosis and fibrosis. Coincidentally, emergent researches have shown that Hes1 may be a novel regulator of ER stress, as shown by its connection with ER stress through UPR pathways [33,34]. In our study, although whether this mechanism could explain the Hes1-regulated ER stress in the kidney after AKI is unclear, we may speculate that there may be a PGC-1α-Hes1-ER stress axis. Although there may be other pathways involved, this study provides useful evidence for further studies to reveal the crosstalk between mitochondrial biogenesis and ER stress. More work should be done to elucidate the detailed mechanisms of the PGC-1α-Hes1-ER stress axis in AKI using siRNA-mediated Hes1 knockdown or Hes1-deficient mice.

It should be noted that there are some limitations in our study. First, since we mainly focused on the early phase after AKI, we only evaluated the effects at 24 h after AKI. Further study should extend these preliminary experiments to at least 72 h to fully understand the effects in the long-term after AKI. Second, we did not examine the effects of ER stress regulation on mitochondrial biogenesis, which may generate a vicious cycle to fully understand the interaction of mitochondrial biogenesis and ER stress in the kidney after AKI. Third, our study was conducted in animal models and more research is warranted to explore the detailed mechanisms in cell culture models.

## Conclusions

In our study, we demonstrated the protective effects of PGC-1α on ER stress in the kidney at 24 h after AKI, which were associated with better mitochondrial biogenesis, modulation of UPR pathway, elevated Mfn2 and decreased Hes1 expression. Our results provide further evidence to understand the crosstalk between mitochondria and ER during AKI and increase the potential for treatment.

## References

[1] Inagi R, Ishimoto Y, Nangaku M. Proteostasis in endoplasmic reticulum-new mechanisms in kidney disease. Nat Rev Nephrol. 2014;10(7):369–378. 24752014 10.1038/nrneph.2014.67

[2] Shpilka T, Haynes CM. The mitochondrial UPR: mechanisms, physiological functions and implications in ageing. Nat Rev Mol Cell Biol. 2018;19(2):109–120. 29165426 10.1038/nrm.2017.110

[3] Lin J, Handschin C, Spiegelman BM. Metabolic control through the PGC-1 family of transcription coactivators. Cell Metab. 2005;1(6):361–370. 16054085 10.1016/j.cmet.2005.05.004

[4] Li SY, Susztak K. The role of peroxisome proliferator-activated receptor γ coactivator 1α (PGC-1α) in kidney disease. Semin Nephrol. 2018;38(2):121–126. 29602395 10.1016/j.semnephrol.2018.01.003 PMC5958619

[5] Tran M, Tam D, Bardia A, et al. PGC-1α promotes recovery after acute kidney injury during systemic inflammation in mice. J Clin Invest. 2011;121(10):4003–4014. 21881206 10.1172/JCI58662 PMC3195479

[6] Tran MT, Zsengeller ZK, Berg AH, et al. PGC1α drives NAD biosynthesis linking oxidative metabolism to renal protection. Nature. 2016;531(7595):528–532. 26982719 10.1038/nature17184 PMC4909121

[7] Pan H, Li JH, Zhou QD, et al. Protective effects of PGC-1α on the blood brain barrier after acute kidney injury. Neurochem Res. 2020;45(5):1086–1096. 32060774 10.1007/s11064-020-02985-5

[8] Puigserver P, Rhee J, Donovan J, , , et al. Insulin-regulated hepatic gluconeogenesis through FOXO1-PGC-1alpha interaction. Nature. 2003;423(6939):550–555. 12754525 10.1038/nature01667

[9] Singh AP, Junemann A, Muthuraman A, et al. Animal models of acute renal failure. Pharmacol Rep. 2012;64(1):31–44. 22580518 10.1016/s1734-1140(12)70728-4

[10] Levey AS, James MT. Acute kidney injury. Ann Intern Med. 2018;168(1):84. 10.7326/L18-0017 29868806

[11] Wu J, Ruas JL, Estall JL, et al. The unfolded protein response mediates adaptation to exercise in skeletal muscle through a PGC-1α/ATF6α complex. Cell Metab. 2011;13(2):160–169. 21284983 10.1016/j.cmet.2011.01.003 PMC3057411

[12] Arensdorf AM, Dezwaan McCabe D, Kaufman RJ, et al. Temporal clustering of gene expression links the metabolic transcription factor HNF4a to the ER stress dependent gene regulatory network. Front Genet. 2013;4:188. 24069029 10.3389/fgene.2013.00188 PMC3781334

[13] Misra J, Kim DK, Choi W, et al. Transcriptional cross talk between orphan nuclear receptor ERRγ and transmembrane transcription factor ATF6α coordinates endoplasmic reticulum stress response. Nucleic Acids Res. 2013;41(14):6960–6974. 23716639 10.1093/nar/gkt429 PMC3737538

[14] Chen X, Zhong J, Dong D, et al. Endoplasmic reticulum stress-induced CHOP inhibits PGC-1α and causes mitochondrial dysfunction in diabetic embryopathy. Toxicol Sci. 2017;158(2):275–285. 28482072 10.1093/toxsci/kfx096 PMC5837255

[15] Yun CW, Han YS, Lee SH. PGC-1α controls mitochondrial biogenesis in drug resistant colorectal cancer cells by regulating endoplasmic reticulum stress. IJMS. 2019; 20(7):1707. 10.3390/ijms20071707 PMC6480203 30959809

[16] de Brito OM, Scorrano L. Mitofusin 2 tethers endoplasmic reticulum to mitochondria. Nature. 2008;456(7222):605–610. 19052620 10.1038/nature07534

[17] Delmotte P, Sieck GC. Endoplasmic reticulum stress and mitochondrial function in airway smooth muscle. Front Cell Dev Biol. 2020;15(7):374. 10.3389/fcell.2019.00374 PMC6974519 32010691

[18] McLelland GL, Goiran T, Yi W, et al. Mfn2 ubiquitination by PINK1/parkin gates the p97-dependent release of ER from mitochondria to drive mitophagy. Elife. 2018;7:e32866. 29676259 10.7554/eLife.32866 PMC5927771

[19] Basso V, Marchesan E, Peggion C, et al. Regulation of ER-mitochondria contacts by parkin via Mfn2. Pharmacol Res. 2018;138:43–56. 30219582 10.1016/j.phrs.2018.09.006

[20] Chen Y, Dorn GW. 2nd. PINK1-phosphorylated mitofusin 2 is a parkin receptor for culling damaged mitochondria. Science. 2013;340(6131):471–475. 23620051 10.1126/science.1231031 PMC3774525

[21] Morris G, Puri BK, Walder K, et al. The endoplasmic reticulum stress response in neuroprogressive diseases: emerging pathophysiological role and translational implications. Mol Neurobiol. 2018;55(12):8765–8787. 29594942 10.1007/s12035-018-1028-6 PMC6208857

[22] Ryu S, Lim W, Bazer FW, et al. Chrysin induces death of prostate cancer cells by inducing ROS and ER stress. J Cell Physiol. 2017;232(12):3786–3797. 28213961 10.1002/jcp.25861

[23] Kristensen CM, Brandt CT, Ringholm S, et al. PGC-1α in aging and lifelong exercise training-mediated regulation of UPR in mouse liver. Exp Gerontol. 2017;98:124–133. 28801170 10.1016/j.exger.2017.08.006

[24] Linkermann A, Chen G, Dong G, et al. Regulated cell death in AKI. J Am Soc Nephrol. 2014; 25(12):2689–2701. 24925726 10.1681/ASN.2014030262 PMC4243360

[25] Yu Y, Sun G, Luo Y, et al. Cardioprotective effects of notoginsenoside R1 against ischemia/reperfusion injuries by regulating oxidative stress- and endoplasmic reticulum stress related signaling pathways. Sci Rep. 2016; 6:21730. 26888485 10.1038/srep21730 PMC4757886

[26] Chen J, Li L, Bai X, et al. Inhibition of autophagy prevents panax notoginseng saponins (PNS) protection on cardiac myocytes against endoplasmic reticulum (ER) stress-induced mitochondrial injury, Ca^2+^ homeostasis and associated apoptosis. Front Pharmacol. 2021;12:620812. 33762943 10.3389/fphar.2021.620812 PMC7982947

[27] Kobayashi T, Kageyama R. Hes1 regulates embryonic stem cell differentiation by suppressing notch signaling. Genes Cells. 2010;15(7):689–698. 20545770 10.1111/j.1365-2443.2010.01413.x PMC2916211

[28] Gupta S, Li S, Abedin MJ, et al. Effect of notch activation on the regenerative response to acute renal failure. Am J Physiol Renal Physiol. 2010;298(1):F209–F215. 19828677 10.1152/ajprenal.00451.2009

[29] Huang R, Zhou Q, Veeraragoo P, et al. Notch2/Hes-1 pathway plays an important role in renal ischemia and reperfusion injury-associated inflammation and apoptosis and the γ-secretase inhibitor DAPT has a nephroprotective effect. Ren Fail. 2011;33(2):207–216. 21332343 10.3109/0886022X.2011.553979

[30] Rani A, Greenlaw R, Smith RA, et al. HES1 in immunity and cancer. Cytokine Growth Factor Rev. 2016; 30:113–117. 27066918 10.1016/j.cytogfr.2016.03.010

[31] Bielesz B, Sirin Y, Si H, et al. Epithelial notch signaling regulates interstitial fibrosis development in the kidneys of mice and humans. J Clin Invest. 2010;120(11):4040–4054. 20978353 10.1172/JCI43025 PMC2964979

[32] Han SH, Wu MY, Nam BY, et al. PGC-1*α* protects from notch-induced kidney fibrosis development. J Am Soc Nephrol. 2017;28(11):3312–3322. 28751525 10.1681/ASN.2017020130 PMC5661291

[33] Li Y, Zhang Y, Fu H, et al. Hes1 knockdown exacerbates ischemic stroke following tMCAO by increasing ER Stress-Dependent apoptosis via the PERK/eIF2α/ATF4/CHOP signaling pathway. Neurosci Bull. 2020;36(2):134–142. 31309426 10.1007/s12264-019-00411-7 PMC6977800

[34] Lee JE, Morrison W, Hollien J. Hairy and enhancer of split 1 (HES1) protects cells from endoplasmic reticulum stress-induced apoptosis through repression of GADD34. J Biol Chem. 2018;293(16):5947–5955. 29491143 10.1074/jbc.RA118.002124 PMC5912459

